# Identification of polycistronic transcriptional units and non-canonical introns in green algal chloroplasts based on long-read RNA sequencing data

**DOI:** 10.1186/s12864-021-07598-y

**Published:** 2021-04-23

**Authors:** Xiaoxiao Zou, Heroen Verbruggen, Tianjingwei Li, Jun Zhu, Zou Chen, Henqi He, Shixiang Bao, Jinhua Sun

**Affiliations:** 1 Institute of Tropical Bioscience and Biotechnology, Hainan Academy of Tropical Agricultural Resource, Chinese Academy of Tropical Agricultural Sciences, Haikou, 571101 Hainan P. R. China; 2 Hainan Provincial Key Laboratory for Functional Components Research and Utilization of Marine Bioresources, Haikou, 571101 Hainan P. R. China; 3 School of BioSciences, University of Melbourne, Parkville, 3010 Australia; 4 Environment and Plant Protection Institute, Chinese Academy of Tropical Agricultural Sciences, Haikou, 571101 Hainan P. R. China

**Keywords:** Chloroplast genome, Polycistronic transcripts, Gene fragmentation, Freestanding ORF, Group II intron, Siphonous algae, PacBio, Iso-seq

## Abstract

**Background:**

Chloroplasts are important semi-autonomous organelles in plants and algae. Unlike higher plants, the chloroplast genomes of green algal linage have distinct features both in organization and expression. Despite the architecture of chloroplast genome having been extensively studied in higher plants and several model species of algae, little is known about the transcriptional features of green algal chloroplast-encoded genes.

**Results:**

Based on full-length cDNA (Iso-Seq) sequencing, we identified widely co-transcribed polycistronic transcriptional units (PTUs) in the green alga *Caulerpa lentillifera.* In addition to clusters of genes from the same pathway, we identified a series of PTUs of up to nine genes whose function in the plastid is not understood. The RNA data further allowed us to confirm widespread expression of fragmented genes and conserved open reading frames, which are both important features in green algal chloroplast genomes. In addition, a newly fragmented gene specific to *C. lentillifera* was discovered, which may represent a recent gene fragmentation event in the chloroplast genome.

With the newly annotated exon-intron boundary information, gene structural annotation was greatly improved across the siphonous green algae lineages. Our data also revealed a type of non-canonical Group II introns, with a deviant secondary structure and intronic ORFs lacking known splicing or mobility domains. These widespread introns have conserved positions in their genes and are excised precisely despite lacking clear consensus intron boundaries.

**Conclusion:**

Our study fills important knowledge gaps in chloroplast genome organization and transcription in green algae, and provides new insights into expression of polycistronic transcripts, freestanding ORFs and fragmented genes in algal chloroplast genomes. Moreover, we revealed an unusual type of Group II intron with distinct features and conserved positions in Bryopsidales. Our data represents interesting additions to knowledge of chloroplast intron structure and highlights clusters of uncharacterized genes that probably play important roles in plastids.

**Supplementary Information:**

The online version contains supplementary material available at 10.1186/s12864-021-07598-y.

## Background

Chloroplasts, the light-harvesting organelles in plants and green algae, were derived from a photosynthetic cyanobacterium through the process of endosymbiosis [1]. Chloroplasts have retained a reduced cyanobacteria-derived genome, which is generally a circular-mapping DNA molecule ca. one hundred to two hundred kilobases in size, although there are exceptions [2, 3]. During endosymbiosis and subsequent genome evolution, most cyanobacterial genes were transferred to the host nucleus or lost, and only a core set of genes encoding key proteins involved in photosynthesis, transcription and translation have been retained in most chloroplast genomes [4].

Chloroplasts have both prokaryotic and eukaryotic properties [5, 6], with gene expression reminiscent of prokaryotes, involving sigma70 promoters and genes organized into operons that are usually transcribed as polycistronic transcripts. Among the eukaryote-like features are the prevalence of introns, highly stable mRNAs, and a more complex regulation of gene expression [7]. Transcripts of chloroplast genes are post-transcriptionally modified in some lineages, including polycistronic transcripts processing, intron splicing, RNA editing and the recently identified non-coding, antisense RNAs and circular RNAs [6–8].

The rapid uptake of high throughput sequencing has led to large numbers of plant and green algal chloroplast genomes being sequenced across the green lineage, advancing our knowledge of their structural diversity and evolutionary dynamics. In the green algal lineage (Chlorophyta), 168 chloroplast genomes are now available on NCBI. The order Bryopsidales, a group of marine seaweeds with a siphonous cell architecture [9], has become a model system for algal chloroplast genome evolution, with studies characterizing a range of genes of possible bacterial origin [10], the evolutionary dynamics of different groups of introns and non-standard open reading frames (ORFs) associated with mobile functions [11], and genome dynamics in relation to habitat features [12].

Bryopsidalean chloroplast genomes also feature a number of genes fragmented into two subsequent ORFs, either with an in-frame stop codon separating them or with a frame shift along the gene, or occasionally more widely spaced around an insertion not associated with group I or group II introns [13]. These genes have previously been considered pseudogenes, but sequence conservation would suggest they are not, and to date it is not known whether and how these genes are transcribed and possibly modified post-transcriptionally. Most of the recent work on green algal chloroplast genomes has been based on short-read sequencing (SRS) data assembled and annotated via largely automated methods, which may in some cases result in incomplete assembly and misannotation, particularly of features like exon-intron boundaries that not transfer well across species. Furthermore, while genome dynamics have been well-characterized, little is known about how genes are transcribed and modified post-transcriptionally.

Here we focused our study on the bryopsidalean species, and use *Caulerpa lentillifera* (Bryopsidales, Chlorophyta), which is an important edible alga with high nutritional and economic values [14–17]. The goal of this study is to fill these knowledge gaps in genomic organization and transcription in bryopsidalean green algae. First, we aim to take advantage of long-read sequencing to assemble a high-quality chloroplast genome, identify potential errors in SRS-based assemblies and evaluate the possibility of structural genome variants. Second, we aim to characterize the expression of polycistronic transcripts and freestanding (non-intronic) ORFs, and evaluate the transcriptional features of fragmented genes using full-length cDNA isoform sequencing (Iso-seq) technologies. Finally, we aim to take advantage of the full-length cDNA sequences to better understand and improve the prediction of exon-intron boundaries in the chloroplast genomes of siphonous green algae.

## Results

### Improved chloroplast genome assembly and annotation

**Fig. 1:**
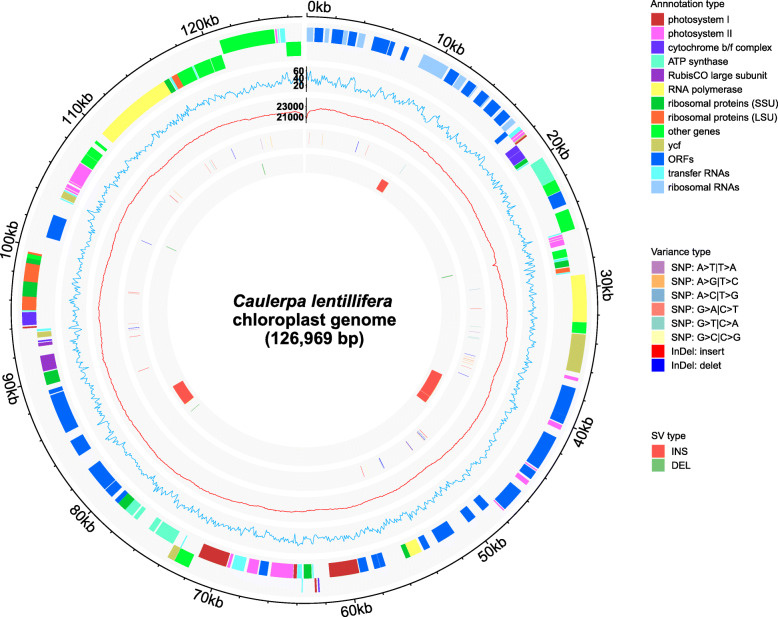
Chloroplast genome map of *Caulerpa lentillifera* (Clcp-v1) and comparison with the previous version, MG753774.1. The outermost circle is positions of Clcp-v1 sequences. Annotation of genes in Clcp-v1 is shown in the second circle, the genes present outside of this circle are transcribed in a clockwise direction, while those insides are transcribed counterclockwise. Genes are colored according to the functional categories listed in the legend. The third and fourth circles indicate the GC content, and mapping depth and coverage of PacBio long-read sequencing data of Clcp-v1, respectively. The fifth and innermost circles indicate the distribution of SNPs and indels, as well as structural variations compared with MG753774.1, respectively. Variation types are colored as shown in the legend

About 3.28 Gbp of PacBio reads from the DNA library of a single genotypic isolate (V1) of *C. lentillifera* showed affinity to chloroplast genome and were assembled. The long reads and high average read coverage (ca. 25,000×), resulted in the chloroplast genome being assembled into a single contig without gaps or ambiguous regions (Fig. 1). The obtained circular chloroplast genome (referred to as Clcp-v1) was 126,969 bp in length.

When compared with the previously reported *C. lentillifera* chloroplast genome (119,402 bp, GenBank Accession No. MG753774.1), the average identity between the sequences is 99.89%, but our assembly is 7.5 kb larger, with 8 structural differences (range from 35 bp to 2.8 kb), 19 InDels and 33 SNVs. Among these differences, several InDels are related to copy number variation of tandem repeat sequences (Fig. 1 and Additional file 1: Tables S1-S3). Analysis of the assembly graph of our long-read DNA sequencing data did not identify any structural heterogeneity, unlike the recent observations for another *Caulerpa* species [18]. The differences between the *C. lentillifera* chloroplast genomes Clcp-v1 and MG753774.1 appear to reflect strain-level variation.

Based on the results of automatic prediction by GeSeq [19] and the PacBio full-length cDNA isoform sequencing (Iso-seq) to guide annotation, a total of 146 genes were annotated in the *C. lentillifera* chloroplast genome, including 76 protein coding genes, 28 tRNA genes, 3 rRNA genes and 39 ORFs (≥300 bp in length) (Fig. 1).

Compared with the annotations in MG753774.1, which was conducted by automated prediction, the gene content of rRNA, tRNA and protein coding genes are almost the same between Clcp-v1 and MG753774.1. However, there are a few differences in gene structural annotation between the two sequences. Most of these differences were caused by incorrect annotation of the exon-intron boundaries, or the annotation of introns where no introns exist. Taking advantages of the full-length cDNA reads, the introns and intron boundaries of rRNA genes and protein-coding genes (such as *rbc*L, *atp*F and *ccs*A) were confirmed, and the misannotated introns in *rps*7, *ycf*20, *cys*A in the previously published genome are corrected in our Clcp-v1 genome (Additional file 2: Figures S1 and S2). *C. lentillifera* has a similar gene content to other *Caulerpa* species, but our re-examination revealed that a few genes were missed in previous studies (Table 1 and Additional file 3: Table S4).

**Table 1:** General characteristics of *C. lentillifera* chloroplast genome and comparison with other *Caulerpa* species

Species	Accession Number ^a^	Length (kb)	Overall GC content (%)	No. of genes			ORFs^c^	Reference
				Protein coding genes ^b^	rRNA genes	tRNA genes		
*C. lentillifera*	This study	126.97	32.43	76	3	28	39	This study
*C. lentillifera*	MG753774.1	119.40	32.68	76 (75)	3	28	45	[20]
*C. manorensis*	NC_037367.1	140.60	34.62	76 (75)	3	28	36	[13]
*C. cliftonii*	NC_031368.1	131.14	37.64	76 (74)^d^	3	27	34	[12]
*C. racemosa*	NC_032042.1	176.52	33.64	76 (75)	3	27	34	[21]
*C. okamurae*	KX809677.1	148.27	34.54	76 (73)^e^	3	27	28	–
*C. verticillata*	NC_039523.1	148.11	33.52	76 (75)^e^	3	28	57	[11]

^a^ Accession numbers refer to sequences in Genbank database at NCBI

^b^ The numbers outside and inside parentheses represent corrected and previously reported gene numbers, respectively. *til*S was included for counting; fragmented genes (such as *rpo*Ba and *rpo*Bb) were counted once

^c^ Open reading frames (ORFs) > = 300 bp were included in the count; Previously annotated ORFs that related to *til*S were excluded. For data consistency, the ORFs of *C. racemosa* (NC_032042.1) and *C. lentillifera* (MG753774.1) were reannotated

^d^ The mis-annotated *pet*L in previous report was included in corrected result of *C. cliftonii*

^e^ The miss annotated *rpl*32 and *pet*L in previous report were included in corrected result of *C. okamurae* and *C. verticillata*

### Chloroplast genes are widely co-transcribed

**Fig. 2:**
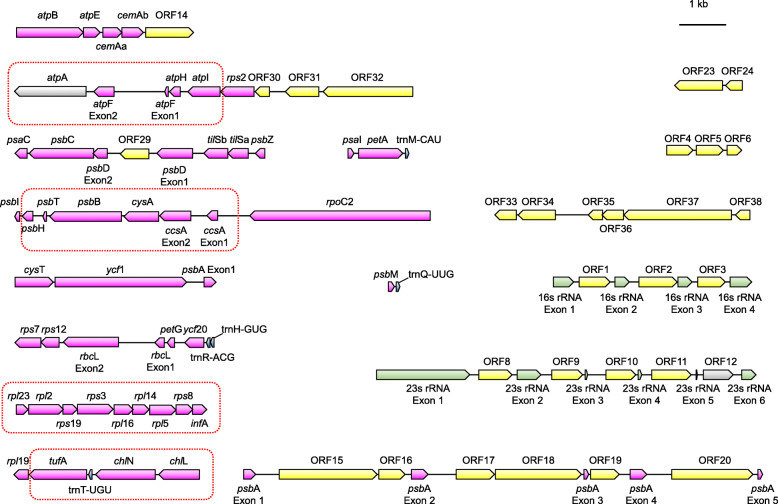
The identified polycistronic transcriptional units (PTUs) in the *C. lentillifera* chloroplast. The protein-coding genes, ORFs, rRNA and tRNA genes are indicated in pink, yellow, green or blue, respectively; *atp*A and ORF12 are shown in gray because the Iso-seq reads did not cover the entire gene. Genes in the dotted boxes are related to the conserved gene clusters across all Bryopsidales identified by Cremen et al. [13]. The gene order, including intronic ORFs within each PTU, is based on the physical genome location

Our full-length Iso-seq data provided evidence for the nature and configuration of polycistronic transcripts in *C. lentillifera*. The 11,048 Iso-seq reads that mapped to the chloroplast covered ca. 87% of the Clcp-v1 genome. Among the 5812 reads covering at least one intact gene/exon and with the same transcriptional direction, 236 cistronic transcriptional types could be identified. Due to these overlapping cistronic transcripts, which may represent different stages of post-transcriptional processing of a polycistronic transcript, we finally concatenated adjacent overlapping cistronic transcripts into the same group, and defined these groups as polycistronic transcriptional units (PTUs). In the full PTU maps, 16 such PTUs covering 43 named protein-coding genes and 29 ORFs were recovered. Among them, the ribosomal protein operon (*rpl*23*-rpl*2*-rps*19*-rps*3*-rpl*16*-rpl*14*-rpl*5-*rps*8-*inf*A) contains the largest number of protein coding genes. Three single gene operons (16S rRNA, 23S rRNA and *psb*A) were considered as PTUs in this study, mainly because they contained a lot of intronic ORFs, and their cistronic RNA seemed to be cleaved into smaller cistrons from our Iso-seq data (Fig. 2 and Additional file 4: Table S5).

Most protein-coding genes joined in a PTU are functionally related and among the 16 PTUs in *C. lentillifera,* four corresponded to previously identified conserved gene clusters across Bryopsidales [13], providing further evidence for the importance of their co-occurrence in PTUs (Fig. 2).

To verify the presence of polycistronic transcripts, four PTUs were validated, including two PTUs of protein-coding genes and two PTUs of ORF clusters. For each PTU, primer pairs spanning from the first to the last gene of the PTU and from the last gene to the next adjacent gene (not part of the PTU) were used. Fragments matching the expected size of each PTU were amplified successfully, confirming the existence of polycistronic transcripts in *C. lentillifera* chloroplast (Additional file 5: Figure S3). In Figure S3b, the two bands of F1/R1 that amplified from cDNA were two isoforms with lengths of 3.5 and 2.7 kb, due to an intron retention event of *rbc*L. In addition, no RT-PCR product was detected for most primer pairs extending beyond the PTUs, suggesting the lack of connection between PTUs and their adjacent genes (Additional file 5: Figure S3), further supporting the classification of PTUs by Iso-seq data. The results of positive RT-PCR amplification of ORF38 ~ *rps*7, and ORF6 ~ ORF7, were in line with expectations, because our Iso-seq data showed that 5′ UTR of some polycistronic transcripts containing ORF38 could span to *rps*7, and 3′ UTR of a few polycistronic transcripts containing ORF6 could span to ORF7 (Additional file 5: Figure S3b, c). However, the weak bands of *ycf*20 ~ *psa*I (Additional file 5: Figure S3b) suggested that the complexity of polycistronic transcription in chloroplast of algae may extend beyond what we inferred from our Iso-seq data.

### Expression of freestanding ORFs

Of the 39 ORFs (24 freestanding and 15 intronic) that we identified in the *C. lentillifera* chloroplast genome, the large majority (35 ORFs) were supported by transcripts in our PacBio Iso-seq data (Additional file 6: Table S6). Similar to other reported bryopsidalean species, most of the ORFs were distributed across the genome but organized into clusters of two or more genes, with the largest one containing 9 ORFs within a 10-kb region. Functional annotation for these ORFs showed that 28 ORFs were found to harbor known structural or functional domains (blastp E-values <1e-10), while 11 ORFs (including 7 novel ORFs that might be specific to *C. lentillifera*) did not show any significant similarity with known proteins in the nr database (Table 2 and Additional file 6: Table S6). Putative homing endonucleases were the most common domains found in the ORFs, including eight LAGLIDADG homing endonuclease, five HNH endonucleases and one GIY-YIG homing endonuclease. Nine ORFs were found to harbor DNA methyltransferase or methylase domains (Table 2).

**Table 2:** Putative function of freestanding ORFs in chloroplast genome of *C. lentillifera*

Putative function		Number of ORFs
Homing endonucleases	LAGLIDADG homing endonuclease	8
	HNH endonuclease	5
	GIY-YIG homing endonuclease	1
Methyltransferase/methylase	N-6 DNA methylase	6
	DNA cytosine methyltransferase	1
	DNA adenine methylase	2
Other function		5
Unknown		11
Total		39

Our Iso-seq data showed that many of these ORFs with unknown function are co-transcribed. For example, of the 9 consecutive ORFs found mentioned above, the last 6 (ORF33-ORF38) were co-transcribed as a single PTU, and the former 3 (ORF30-ORF32) were co-transcribed along with three ATP synthase genes and *rps*2 (Fig. 2 and Additional file 4: Table S5).

### Expression of fragmented genes

**Fig. 3:**
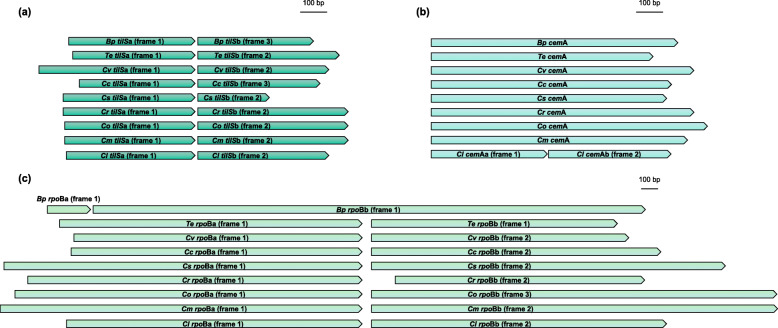
Alignments of the fragmented genes. Fragmentation of *til*S (**a**), *cem*A (**b**), and *rpo*B (**c**) in *Caulerpa* species are shown. *Tydemania expeditionis* (*Te*) and *Bryopsis plumosa* (*Bp*) were selected as representatives of suborder Halimedineae and Bryopsidineae, respectively. *Cv*: *C. verticillata, Cc*: *C. cliftonii, Cs*: *C. serrulata, Cr*: *C. racemosa, Co*: *C. okamurae, Cm*: *C. manorensis, Cl*: *C. lentillifera*

Because previous studies had revealed that two protein coding genes, RNA polymerase b-subunit (*rpo*B) and tRNA (Ile)-lysidine synthase (*til*S) were fragmented in several bryopsidalean chloroplast genomes, we wanted to investigate whether these were transcribed and possibly post-transcriptionally modified. In addition to *til*S and *rpo*B, our *C. lentillifera* genome showed a third fragmented gene, the chloroplast envelope membrane protein (*cem*A) which was also supported by the previous sequenced *C. lentillifera* MG753774.1. While *rpo*B and *til*S were fragmented into two pieces across all the published *Caulerpa* species, *cem*A fragmentation was only observed in *C. lentillifera*. Frame shifts were present in all these genes in *Caulerpa* species, leading them to be divided into two adjacent ORFs (labeled as a and b in this study), respectively (Fig. 3).

No single PacBio RNA read covered the entire region encompassing *rpo*Ba and *rpo*Bb, but a number of reads covered either only *rpo*Ba, or from *rpo*Bb (5′ truncated) to *cys*T/*cyf*1, suggesting that *rpo*Ba and *rpo*Bb are probably translated from different mRNA molecules. The subunits of the two other fragmented genes (*cem*Aa and *cem*Ab, *til*Sa and *til*Sb) were observed to be co-transcribed in PTUs, but some shorter reads (some are 5′ or 3′ truncated) covered either fragment a or b, indicating that they might also be cleaved into different transcripts during the post-transcriptional process (Additional file 7: Figure S5). We were unable to detect Shine-Dalgarno (SD)-like sequences (a sequence located upstream of the start codon to initiate translation [22, 23]) in the 5′ untranslated region of any of the shorter transcripts, so it is unclear whether the two subunits could be separately translated. To verify whether post-transcriptional RNA editing may be used to overcome the frame shifts within the fragmented genes, we carefully compared the sequences between chloroplast genome and the aligned Iso-seq reads. No RNA editing site was found, suggesting it is unlikely that these fragmented genes are restored to a single continuous reading frame by RNA editing.

**Fig. 4:**
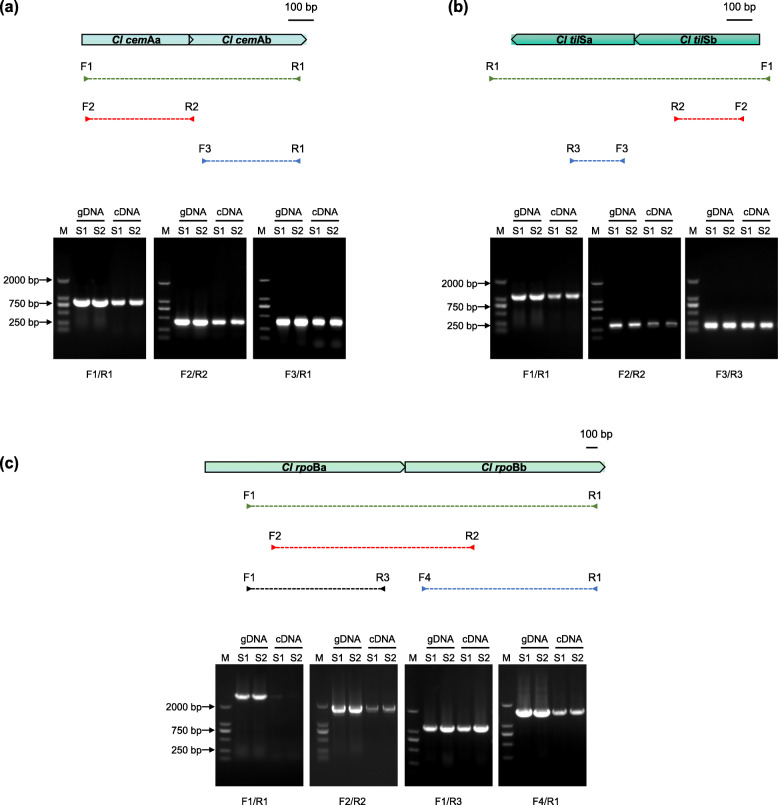
Transcriptional characteristics analysis of fragmented genes by RT-PCR, *cem*A (**a**), *til*S (**b**), *rpo*B (**c**). The positions of primer pairs are shown as arrows with different color. Electrophoresis of PCR products on 1% agarose gel. gDNA (genomic DNA) was used as positive control. S1 and S2 are two different individuals of *C. lentillifera.* Full-length blots/gels are presented in Supplementary Figure S6

Because the RNA library for Iso-seq was constructed by polyA-enrichment, which may not fully reflect the transcriptional state of chloroplast genes, we further validated the transcription patterns of the three fragmented genes by RT-PCR. In accordance with Iso-seq results, the two pieces of *cem*A and *til*S were confirmed to be co-transcribed. There was extremely weak amplification of the *rpo*Ba to *rpo*Bb section, but the bands representing amplicons of separate *rpo*Ba or *rpo*Bb fragments were very strong in comparison. This suggests that while the two pieces of *rpo*B could occasionally be co-transcribed, most transcripts exist as *rpo*Ba and *rpo*Bb separately (Fig. 4).

### Atypical group II introns with widely conserved features

**Fig. 5:**
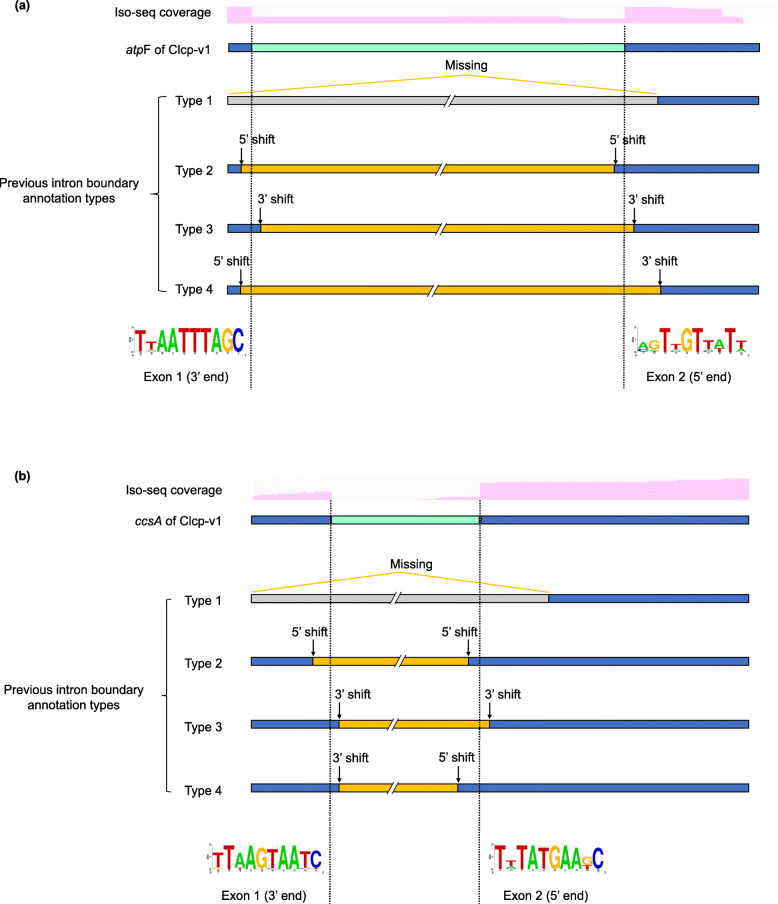
Comparison conserved intron positions after adjusting with previous exon-intron boundaries annotation types. Comparison exon-intron boundaries of *atp*F (**a**) and *ccs*A (**b**). The yellow and grey boxes indicate the position of previously annotated introns and mis-annotated gene portions in current databases, respectively. Sequences logos at the 3′ boundary of exon 1 and 5′ boundary of exon 2 in *atp*F and *ccs*A, respectively, are shown at the lower part

Automated predictions (MFannot and Geseq) and PacBio Iso-seq guided annotation resulted in a few clear differences of predicted introns and exon-intron boundaries (Table 3). For example, our Iso-seq data supported that *atp*F and *ccs*A both contained introns, but annotation programs did not predict these introns. Further comparative analysis of *atp*F and *ccs*A homologues in Bryopsidales found that these gene models were more often missed or incapable annotated accurately if they harbored introns. Thus, we collected these genes across Bryopsidales (40 *atp*F and 39 *ccs*A sequences) and tried to manually reannotate them using our Iso-seq-informed intron boundaries as a reference (Additional file 8: Table S7). As a result, in the 39 published *atp*F genes with introns, intron boundaries had to be adjusted in 36. For *ccs*A, of the 36 instances, 35 intron boundaries were incorrect. Interestingly, after adjusting the exon-intron boundaries of *atp*F and *ccs*A in these species, the reading frame of all these genes lined up very well and the amino acids sequences near the exon-intron boundaries showed much stronger conservation, with the sequence identities of amino acids (calculated for the entire gene) improving from 50.42 to 54.37% for *atp*F and from 42.90 to 47.83% for *ccs*A (Additional file 9: Figures S7-S8). Furthermore, the analyzed introns seemed to occupy the same positions across the Bryopsidales, and the conservation could be also supported by intron-less *atp*F or *ccs*A in other species of Bryopsidales, and even in other class of Chlorophyta (Fig. 5 and Additional file 9: Figures S7-S8).

**Table 3:** Comparison of intron annotation between automated predictions and Iso-seq alignment of chloroplast genes in *C. lentillifera*

Intron contained genes	Number of introns			Putative intron type ^c^
	Iso-seq data	FMannot	Geseq	
rrn16	3	0	0	Intron 1: undetermined (LHE) Intron 2: undetermined (LHE) Intron 3: undetermined (LHE)
rrn23	5	0	0	Intron 1: group I Intron 2: group I Intron 3: group I Intron 4: group I Intron 5: undetermined (LHE)
*psb*A	4	4	4^a^	Intron 1: group II Intron 2: group II Intron 3: group I Intron 4: group II
*psb*D	1	1	1	Intron 1: group I
*rbc*L	1	1	1	Intron 1: group II
*atp*F ^b^	1	0	0	Intron 1: non-canonical group II (derived domain V)
*ccs*A ^b^	1	0	0	Intron 1: non-canonical group II
*rpo*C2	0	0	1	–
*rpo*C1	0	0	1	–

^a^ Boundaries of *psb*A intron 2 cannot be accurately predicted

^b^ *atp*F and *ccs*A were mis-predicted by FMannot and Geseq

^c^ Introns of *atp*F and *ccs*A were classified as non-canonical group II intron here, because they have conserved or derived domain V but lack of typical exon-intron boundaries and intronic ORFs of group II intron. LHE indicates a LAGLIDADG homing endonuclease domain within the intron

**Fig. 6:**
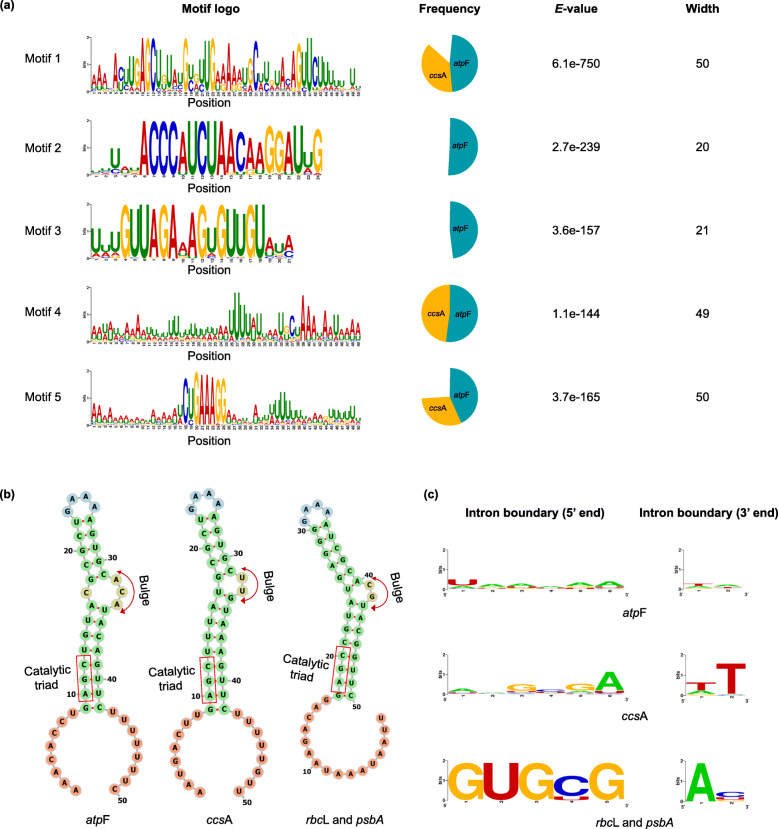
Motif discovery and distinct features of the introns from *atp*F and *ccs*A**.** The intron features were comparison with other group II introns (mainly from *rbc*L and *psb*A) in the Bryopsidales. **a** The top five motifs that were found from the 40 *atp*F and 37 *ccs*A intron sequences by MEME. Height of letters in motif logos indicates the occurrence of nucleotides at specific position. The frequency pie chart represents the frequency of occurrences of each motif across the 77 intron sequences, and the *E*-value indicates the statistical significance of the motifs. Width represents the number of nucleotides in a particular motif. **b** Comparison of the consensus secondary structures of domain V among *atp*F *ccs*A, and other group II introns (*rbc*L and *psb*A). The conserved trinucleotide 5′-AGC-3′, and the 2-nt bulge in domain V, which were the two catalytically important sites of group II introns, were highlighted by boxes and double arrow curves. **c** Comparison of the intron boundaries (5′ and 3′ end) among *atp*F *ccs*A, and other group II introns (*rbc*L and *psb*A)

At least five motifs were identified in the 40 *atp*F and 37 *ccs*A intron sequences with high statistical support (E-value < 10^− 140^). Among them, motifs 1, 4 and 5 were the most common (Fig. 6a and Additional file 10: Figure S9), with motif 1 located towards the 3′ end of almost all of these introns, and motif 5 located upstream of motif 1, with the average interval between these two motifs 63 bp (Additional file 10: Figure S9). Motif 5 was composed of a highly conservative element (CYGAAAGG) and AT-rich flanking sequence.

Intron type prediction showed that most of the introns in *atp*F or *ccs*A are putative group II introns, similar to most other introns of protein coding genes in the chloroplast genome of *C. lentillifera* (Table 3 and Additional file 8: TableS7). Secondary structure analysis showed that motif 1 overlapped with intron domain V (most were derived type) and includes the highly conserved 5′-AGC-3′ trinucleotide (Fig. 6b).

However, there are several distinct features of the introns in *atp*F and *ccs*A in comparison with other group II introns (mainly compared with those of *psb*A and *rbc*L here) in Bryopsidales. Firstly, intron boundaries of *ccs*A and *atp*F were highly variable, while the more canonical group II introns of *psb*A and *rbc*L had conserved boundary nucleotide patterns (5′ GUGYG … AY 3′, Fig. 6c). Secondly, the bulge in domain V, which is another catalytically important site conserved among most group II introns is much more variable in *atp*F and *ccs*A introns (Fig. 6b and Additional file 10: Figure S10); Thirdly, although intronic ORF that contains a reverse transcriptase (RT) and/or intron maturase (IM) domain are common in group II introns of the Bryopsidales [13], none of the 86 intronic ORFs (> = 150 bp) in *atp*F and *ccs*A were predicted to contain conserved domains of known function (Additional file 11: Table S8).

## Discussion

Our PacBio long read experiment was originally designed to characterize the nuclear genome and transcriptome of *C. lentillifera*. Because a substantial fraction of PacBio reads from the DNA and RNA libraries were of chloroplast origin, we took advantage of these data to study chloroplast genome organization and transcription. Using long-read sequencing, we obtained an intact chloroplast genome and a well-defined gene structural annotation of *Caulerpa lentillifera*. The new genome is 7.5 kb larger than the previously reported genome sequence [20], and in addition to SNVs and a few possible structural variations between the two versions, there are several indels relating to copy number variation of tandem repeat sequences (Additional file 1: Table S2). Plastome structural variations in green algae have been identified in the Streptophyta [24], even within the same species, such as *Capsosiphon fulvescens* [25]. As the sample of MG753774.1 was collected from Lingtou sea aera of Hainan, China [20], while the sample of this study was an offspring of strains originated from Nha Trang, Vietnam, these differences could be due to intraspecific differences between the isolates. However, limitations of using only short sequence reads in the previous work may have also contributed to the differences, as short reads can fail to assemble repetitive sequences [26]. Since the raw reads for the previously published genome are not available, we cannot determine the exact reason for the differences between the two sequences at present. Long-read sequences also permit identifying heteroplasmy within individuals, as recently shown in the chloroplast genome of a related species by nanopore sequencing [18]. Our PacBio long-read data did not reveal any evidence of such structural variations, and in our opinion the prevalence and nature of heteroplasmy across the siphonous green algae requires further work based on long-read methods that deliver highly accurate reads.

Several bioinformatic tools for automated feature annotation of chloroplast genomes have been developed, but relatively little work has been done to compare their predictions to experimentally determined RNA sequences. Our Iso-seq work shows that the majority of genes encoding proteins and rRNA were accurately predicted by MFannot and GeSeq. However, we found that the Iso-seq data-guided annotation could greatly improve the annotation of introns and exon-intron boundaries. Taking our exon-intron boundary information as a reference, we were able to greatly improve structural annotations of *atp*F and *ccs*A across the Bryopsidales, and corrected intron structures facilitated by the analysis of the unusual characteristics of these introns. Several common features of the *atp*F and *ccs*A introns were identified, such as the domain V motif and other common motifs upstream from it. Domain V, which is one of the six conserved domains radiating from a “central wheel” of group II introns, is the most conserved element and important component in catalytic reactions of group II introns [27, 28]. It was clear that the 2-nt bulge (AY) and the catalytic triad (AGC or CGC for some introns) at the stem of domain V are most important for chemical catalysis of excision [29, 30]. Although the catalytic triad are still conserved retained across all the analyzed group II introns of Bryopsidales, the bulge of domain V in *atp*F and *ccs*A introns are relative variable, indicating the splicing mechanisms of these introns might be different from typical group II introns. Previous work mainly based on land plants and *Euglena* showed that most group II introns are degenerated in their RNA structures or have lost the intron encoded proteins [31]. Our results indicate that the introns in *atp*F and *ccs*A have several obvious differences from canonical group II introns, including the absence of consensus intron boundary sequences, ORFs lacking homology to splicing or mobility, and deviant overall structure making it difficult to accurately determine the secondary domains other than domain V. However, our Iso-seq data showed that the introns in *atp*F and *ccs*A were spliced predictably, suggesting that an effective mechanism has evolved to recognize and splice these atypical introns in bryopsidalean chloroplasts.

The fragmentation of several protein-coding genes has been a puzzling feature of green algal chloroplast genomes. In this study, three protein-coding genes were found to be fragmented in the *C. lentillifera* plastid genome, with *cem*A shown to be fragmented in addition to the previously reported *til*S and *rpo*B, which are known to be fragmented across Bryopsidales [13] and some other green algal lineages (e.g. [29, 32, 33]. Considering that *cem*A is not fragmented in other *Caulerpa* species, it likely represents a recent event. This observation, along with reports of some other fragmented genes such as *rpo*C1 and *rpo*C2 in *Chlamydomonas* species [34], suggests that gene fragmentation may be fairly common in green algal chloroplast genomes. The fragmented genes in *Caulerpa* retained high sequence conservation following the fragmentation, a clear indication that they are not pseudogenes. Our Iso-seq data and RT-PCR results provide clear evidence for transcription of these genes. They also indicate that the two pieces of both *cem*A and *til*S are co-transcribed in transcriptional units, but the presence of shorter transcripts covering either fragment of these genes suggests that the transcripts may be divided into two portions by RNA processing mechanisms. Our results for *rpo*B contrast with the other genes, rather showing that while the two fragments were occasionally found on a single transcript, they were more commonly transcribed separately. A careful comparison between chloroplast genome and the aligned Iso-seq reads showed no evidence for RNA editing, thus it seems unlikely that the frame shifts in these fragmented genes were modified to restore normal reading frames. Ribosomal frameshifting [35] could be a hypothetical alternative mechanism to correct the frameshifts in fragmented genes at the level of translation, but the fact that various types of gene fragmentation exist in Bryopsidalean lineages [13], including some with longer inserts between the fragments, would suggest this is unlikely and that it is more likely that the two pieces of these fragmented genes are translated separately and combine after translation. We did not find SD-like sequences (translation initiation signals of bacteria and some chloroplast mRNAs) upstream of the translation initiation sites of the gene fragments, so it remains to be confirmed whether the transcriptional products of fragments a and b are separately translated and perform their normal functions by forming protein complexes of both subunits. Nevertheless, gene fragmentation (or gene fission) as well as gene fusion are important mechanisms that contribute to the evolution of gene architecture and origination of new genes. Gene fusion/fission was major contributor to evolution of multi-domain proteins in bacteria and creation of new genes in *Drosophila* [36, 37], and the mechanism of the origin of gene fission has been revealed as a two-step process consisting of duplication and degeneration in *Drosophila.* Recently, gene fragmentation was found to be very prominent in mitochondrial genomes of Diplonemids, where the resulting modules (gene fragments) are transcribed separately, which might contribute to a gradual increase in the complexity of a given cellular machinery [38]. What drives gene fragmentation in chloroplast genomes as well as the mechanisms and consequences of this process in these organelles remain open questions.

Our Iso-seq data allowed us to experimentally verify polycistronic mRNAs and post-transcriptional isoforms, which are important for understanding the mechanisms of plastid genome expression. Although transcriptional and post-transcriptional regulation of chloroplast genes have been well studied in higher plants [6, 39–42], little is known about the situation in algae. Earlier studies suggested that, unlike the situation in higher plants, polycistronic gene expression is unlikely in chloroplast of the green alga *Chlamydomonas reinhardtii*, because its plastome has much larger intergenic spacer regions which consist of numerous short dispersed repeats [43]. However, recent studies indicated that several chloroplast genes may be co-transcribed by examining the uninterrupted coverage of RNA-seq data [44, 45], suggesting transcript processing in chloroplasts of green algae may be as important as in plants. Moreover, a latest study have even discovered a number of polycistronic gene expression examples in nuclear genome of two divergent green algae species, suggesting their biological importance in the green algal lineage [46].

Previous studies showed that polycistronic transcripts can be transcribed using multiple promoters, and numerous transcriptional start sites have been identified within operons. It is also reported that chloroplast genes are typically organized into polycistronic transcription units that give rise to complex sets of overlapping RNAs through a series of processing steps [7, 47–49]. Similarly, several overlapping cistronic transcripts with various initiation and termination positions or representing different processing stages were found in our Iso-seq data. In this study, through a combination of Iso-seq analysis and RT-PCR verification, we revealed that more than half of the protein-coding genes are co-transcribed with adjacent genes, forming polycistronic transcripts of up to 9 genes in *Caulerpa*. Among the 16 defined PTUs, seven were supported by Iso-seq reads covering the entire PTU. Two (*atp*B-*atp*E-*cem*A-ORF14, *rps*7-*rps*12-*rbc*L-*pet*G-*ycf*20) of the remaining 5 PTUs had only the first or last gene incompletely covered by Iso-seq reads and have been verified by our RT-PCR experiment (Additional file 5: Figure S3). The final 4 PTUs were very long (> 5.3 kb), and although overlapping Iso-seq reads suggested they form a continuous PTUs, we had no single Iso-seq read covering these PTUs completely. Because two (23S rRNA and *psb*A operons) of these large PTUs were consisted of a single gene and their respective intronic ORFs, we thought this phenomenon might either due to the full-length polycistronic transcripts being recovered as truncated Iso-seq reads, or the relatively low throughput of long-read sequencing resulting in insufficient detection of very large (or rapidly processed) primary transcripts.

In addition, because we used very strict criteria to consider genes as co-transcribed on PTUs, several genes flanking our PTUs but that were not entirely covered by Iso-seq reads were not counted as part of the PTUs, so the extent of gene co-transcription is probably even larger. The unexpected positive RT-PCR amplification of *ycf*20 ~ *psa*I (Additional file 5: Figure S3b) also suggested that polycistronic transcription in chloroplast of algae may be more complex than what detected from our Iso-seq data.

Our work, along with observations of co-transcribed chloroplast gene clusters in *C. reinhardtii* based on RNA-seq coverage analysis [44, 45], provide clear evidence for polycistronic transcription in algae. Unsurprisingly, genes on the same PTU in *C. lentillifera* were often functionally related. This observation extends to the co-transcribed clusters of unknown ORFs, which often shared conserved functional domains of the same class within the PTU (Additional file 4: Table S5). Four of the six conserved gene clusters in Bryopsidales [13] were found to be co-transcribed in PTUs, underlining the strong evolutionary conservation of co-transcription and the importance of the co-occurrence of these genes. The remaining two conserved gene clusters observed in Bryopsidales (*psa*M-*psb*30-*psb*K-*psb*N-*trn*M and *psb*E-*psb*F-*psb*L-*psb*J) were not observed as PTUs in this study. This is because our full-length RNA data did not contain any reads of the genes in question, perhaps due to their lower levels of expression or faster degradation.

Iso-seq has been successfully applied in the discovery of polycistronic transcripts and investigation of complex post-transcriptional processes in fungi, viruses, plants and green algae [46, 50–52], and in classification of chloroplast operons in duckweed [53]. For our application in the chloroplast, we were limited by the current sequence library construction technology of Iso-seq based on a modified oligo (dT) primer (CDS Primer IIA) for reverse transcription of polyA+ tail transcripts to generate full length cDNA of nuclear genome. This is likely to reduce chloroplast transcripts, because organelle mRNA may not be polyadenylated to the same degree as nuclear-encoded genes, and poly-adenylation of organellar transcripts may also contribute to mRNA degradation [54–57]. As the number of organellar mRNA might not be captured by Iso-seq libraries, and the detected transcripts may therefore not reflect their abundance in the cell, we did not attempt quantitative work on PTU expression here. Combining rRNA-depleted RNA-seq and Iso-seq data, or using native direct RNA sequencing technology (such as the Oxford Nanopore technology with the ultra-long reads) may help to overcome these problems for further chloroplast gene expression studies.

## Conclusion

Our study is the first to experimentally determine and examine the genomic organization and transcriptional units in green algae using long-read sequencing technology, providing new insights into structural variations, expression of polycistronic transcripts, freestanding ORFs and fragmented genes in algal chloroplast genomes. Drastic improvements in the detection of exon-intron boundaries using Iso-Seq data permitted a detailed investigation of the structural annotation of intron-containing genes across Bryopsidales, revealing atypical Group II introns with distinct features and conserved positions in the *atp*F and *ccs*A genes. Our results also further our knowledge of gene fission in chloroplast genomes and form a valuable resource for further organellar transcriptomics studies.

## Methods

### Nucleic acid isolation and sequencing

A single genotypic isolate (V1) of *C. lentillifera,* which was an offspring of strains originated from Nha Trang, Vietnam, was collected from our marine culture base in Changjiang, Hainan province and then cultured in sterilized seawater. To minimize the contamination of environmental microbes, the thallus of V1 was treated with a combination of various antibiotics following Brawley et al. [58] for 2 weeks.

For genome sequencing, DNA of the *C. lentillifera* isolate V1 was extracted using a Plant Genomic DNA Kit (DP305, Tiangen Inc., Beijing, China) following the manufacturer’s instructions. A 20-kb insert SMRTbell library was prepared and sequenced by Novogene (Beijing, China) using the PacBio Sequel platform (Pacific Biosciences, CA, USA).

For PacBio full-length cDNA isoform sequencing (Iso-Seq), *C. lentillifera* samples were treated under multiple conditions, such as high temperature (30 °C), low temperature (18 °C), high salinity (50 PSU), low salinity (15 PSU), high light (260 μmol photons·m^− 2^·s^− 1^), shading, desiccation and normal conditions (25 °C, 32PSU and 10 μmol photons·m^− 2^·s^− 1^). Total RNA of each treatment was isolated with the RNAprep pure plant kit (Tiangen Inc., Beijing, China) and treated with RNase-free DNaseI (RT411, Tiangen Inc., Beijing, China) following the manufacturer’s instructions. The quality of RNA was checked on a Bioanalyzer 2100 system (Agilent, Palo Alto, CA, USA). Then extracted RNAs were pooled evenly, and Iso-Seq libraries were constructed with SMARTer™ PCR cDNA Synthesis Kit (Clontech, CA, USA) according to the manufacturer’s instructions, which uses a modified oligo (dT) primer (CDS Primer II A) for reverse transcription of polyA+ tail transcripts. cDNA size was selected by BluePippin™ Size-Selection System (Sage Science), and then sequenced on the PacBio Sequel platform.

### Chloroplast genome assembly

The PacBio raw reads were assembled by Falcon (pb-assembly 0.0.8) [59]. From this draft assembly, seven contigs which had high sequence similarity with chloroplast genomes of *Caulerpa* were identified as candidate chloroplast sequences by blastn using related chloroplast genomes as query. The PacBio raw reads were mapped to the draft assembly by minimap2 [60] and all reads mapping with less than 0.2 divergence and alignment length exceeding 2000 bp were selected for de novo assembly in Canu 1.9 [61] with parameters corOutCoverage = 5000, genomeSize = 150 k, rawErrorRate = 0.200, correctedErrorRate = 0.035, minReadLength = 20,000, minOverlapLength = 8000. As a result, a single contig was obtained, and inspected by using Bandage [62] to evaluate the presence of structural variations in the assembled genome. Finally, the obtained contig was polished with arrow and pilon by using default parameters, and set as a circular molecule with the start point at the beginning of the 16S rRNA gene. The obtained circular *C. lentillifera* chloroplast genome is referred to as Clcp-v1. Then, the de novo assembled Clcp-v1 sequence was compared with the previously reported *C. lentillifera* chloroplast genome (NCBI GenBank accession number: MG753774.1).

### Chloroplast genome annotation and exon-intron boundaries

Initial annotations of *C. lentillifera* chloroplast DNA were performed by GeSeq [19] using the published chloroplast genomes of *Caulerpa* species (NCBI GenBank accession numbers: NC_037367.1, NC_032042.1, KX809677.1, NC_031368.1, NC_039523.1 and MG753774.1) as BLAT reference sequences [11–13, 20, 21], and selected the options to perform tRNAscan-SE v2.0.6 and ARAGORN v1.2.38 to detect tRNA genes, and HMMER profile search to detect chloroplast CDS and rRNA. ORFs with a minimum size set at 300 bp were identified using ORFfinder from NCBI.

Then, the predicted rRNA genes, tRNA genes, protein-coding genes, putative open reading frames and additional features such as the exon-intron boundaries of intron-containing genes, were compared manually to our PacBio Iso-seq data and modified where necessary. The generated chloroplast genome sequence of *C. lentillifera* in this study is available in GenBank under accession number MT271684, and all related PacBio raw data have been deposited on the Sequence Read Archive (SRA) database of NCBI with the number PRJNA658421.

Genes for which predicted exon-intron boundaries differed from those based on Iso-seq data (mostly *atp*F and *ccs*A) were investigated in more detail, intron classification was predicted by using RNAweasel [63], RFAM database searches [64], and the BLAST of conserved domains encoded by predicted ORF, and followed by manual inspection. Because RNAweasel, which is based on RNA secondary structure profiles, and RFAM database searches both gave similar results, suggesting that most introns of *atp*F and *ccs*A are putative Group II introns with conserved or derived domain V, in order to compare intron features of *atp*F and *ccs*A to other group II introns, two additional protein coding genes (*rbc*L and *psb*A) harboring group II introns in *C. lentillifera* were also analyzed. Additional sequences of these four genes across the Bryopsidales were sourced from Genbank. The exon-intron boundaries were compared through multiple sequence alignment, and corrected manually by referring to the intron boundary information of *atp*F, *ccs*A, as well as *rbc*L and *psb*A of *C. lentillifera* supported by our Iso-seq data. Identification of motifs in intronic sequences was performed by MEME [65], in addition, in order to detect more intronic ORFs that may relate to splicing or mobility of the group II introns in *atp*F and *ccs*A, the minimum size of ORF predictions was set at 150 bp. Secondary structure predictions of relative intronic sequences from *atp*F, *ccs*A and other genes that contained group II introns were performed by RNAfold [66]. Consensus secondary structures for the alignments of group II introns from these genes, were carried out by RNAz [67] based on both thermodynamic stability and structural conservation.

### Prediction of polycistronic transcripts

Our full-length Iso-seq transcripts were mapped to Clcp-v1 with Gmap [68], with parameters --no-chimeras-cross-species--expand-offsets 1-B5-K50000-f samse-n 1. To avoid artifactual chimeric transcripts, we just used Full-length Non-Chimeric (FLNC) reads for PTU analysis without clustering or assembling them. Polycistronic transcripts, introns and RNA editing sites were identified through the mapped data and visualized using IGV (Integrative Genomics Viewer, version 2.5.2, https://software.broadinstitute.org/software/igv/download).

To calculate how many types of co-transcribed genes can be classified in *C. lentillifera* chloroplast genome, we detected gene clusters (two or more adjacent genes) oriented in the same direction and occurring together in at least one Iso-seq long read. To increase credibility of the results, we used even stricter criteria: only those genes (or exons of intron-containing genes) completely covered by PacBio Iso-seq reads with the same transcriptional direction, were considered as part of a cistronic transcriptional type. Then the adjacent and overlapping cistronic transcripts, which may represent different stages of post-transcriptional processing of a polycistronic transcript, were combined into full PTU maps.

### RT-PCR validation of polycistronic transcripts and fragmented gene expression

To validate the existence of polycistronic transcripts and further evaluate the hypothesis that both pieces of some fragmented genes (*rpo*Ba and *rpo*Bb, *til*Sa and *til*Sb, *cem*Aa and *cem*Ab) were transcribed on a single mRNA, we carried out RT-PCR experiments. Total RNA samples were isolated using E.Z.N.A. Plant RNA Kit (R6827–01, OMEGA Bio-tek, GA, USA), and contaminated genomic DNA was removed with RNase-Free DNase I Set (E1091–01, OMEGA Bio-tek, GA, USA) according to the manufacturer’s instructions. cDNAs were synthesized with random hexamer primers by using the RevertAid First Stand cDNA Synthesis Kit (K1622, Thermo Scientific, MA, USA). To verify polycistronic transcripts, primer pairs were designed to span the whole PTU, or from each end of the PTU to their adjacent genes. For fragmented gene expression validation, gene specific primers were designed to cover different region of the fragmented genes. All the primers used in this study were designed by using Primer Premier (version 5.0, Premier Biosoft International, USA), and are listed in Additional file 12: Table S9.

## Supplementary Information

**Additional file 1: Table S1.** Structural variations between Clcp-v1 and MG753774.1. **Table S2.** Indels between Clcp-v1 and MG753774.1. **Table S3.** Single nucleotide variants (SNVs) between Clcp-v1 and MG753774.1.

**Additional file 2: Figure S1.** Confirmation of exon and intron boundaries of intron-containing genes in chloroplast genome of *C. lentillifera*. **Figure S2.** Correction of misleading introns in chloroplast genome of *C. lentillifera*.

**Additional file 3: Table S4.** Comparison of chloroplast genes among members of *Caulerpa*.

**Additional file 4: Table S5.** Polycistronic transcriptional units and their relative transcriptional isoforms in chloroplast genome of *C. lentillifera*.

**Additional file 5: Figure S3.** RT-PCR validation of four PTUs in chloroplast genome of *C. lentillifera*. Full-length blots/gels are presented in Supplementary Figure S4. **Figure S4.** The uncropped full-length gel for RT-PCR validation of PTUs in Figure S3.

**Additional file 6: Table S6.** BlastX results and conserved domains of ORFs in chloroplast genome of *C. lentillifera*.

**Additional file 7: Figure S5.** Iso-seq reads alignment of the three fragmented gene in chloroplast genome of *C. lentillifera*. **Figure S6.** The uncropped full-length gels for transcriptional characteristics analysis of fragmented genes in Fig. 4.

**Additional file 8: Table S7.** List of intron distribution and intron type analysis of *atp*F and *ccs*A in Bryopsidales.

**Additional file 9: Figure S7.** Comparison of multi-sequence alignments between original annotation sequences in the database (a) and amino acid sequences that deduced by adjusting the exon-intron boundaries (b) of *atp*F. **Figure S8.** Comparison of multi-sequence alignments between original annotation sequences in the database (a) and amino acid sequences that deduced by adjusting the exon-intron boundaries (b) of *ccs*A (partial).

**Additional file 10: Figure S9.** Locations of the top five motifs identified from the 40 *atp*F and 37 *ccs*A intron sequences. Motif 1 to 5 were represented as block diagrams with different colors as shown in the figure. **Figure S10.** Sequence logos of domain V from *atp*F, *ccs*A, and group II introns (*rbc*L and *psb*A), respectively. The two catalytically important elements (the catalytic triad and the bulge region) in domain V were highlighted.

**Additional file 11: Table S8.** Prediction of intronic ORFs of *atp*F and *ccsA* in Bryopsidales.

**Additional file 12: Table S9.** List of primers used for RT-PCR validation.

## Data Availability

The datasets supporting the findings of this article are available in the Sequence Read Archive (SRA) database of NCBI with the number PRJNA658421 (https://www.ncbi.nlm.nih.gov/bioproject/?term=PRJNA658421). The generated chloroplast genome sequence of *C. lentillifera* in this study is available in GenBank under accession number MT271684. The reference datasets (NC_037367.1, NC_032042.1, KX809677.1, NC_031368.1, NC_039523.1, MG753774.1) for assembly or initial annotations of chloroplast genome of *C. lentillifera* (Clcp-v1) were obtained from Genbank at National Center for Biotechnology Information (NCBI) database. All the accession numbers listed in Table 1, and Supplementary Tables S6, S7, and S8, were searched and retrieved from nucleotide or non-redundant proteins database at NCBI.

## Abbreviations

BLAST: Basic local alignment search tool
PacBio: Pacific Biosciences
Iso-Seq: Isoform sequencing
PTU: Polycistronic transcriptional unit
