# Supplementary material for: Identification of polycistronic transcriptional units and non-canonical introns in green algal chloroplasts based on long-read RNA sequencing data
Source: BMC Genomics. 2021 Apr 23;22:298. doi: 10.1186/s12864-021-07598-y (PMC8063479; doi:10.1186/s12864-021-07598-y)
Supplement: Supplementary file 1 — Additional file 1: Table S1. Structural variations between Clcp-v1 and MG753774.1. Table S2. Indels between Clcp-v1 and MG753774.1. Table S3. Single nucleotide variants (SNVs) between Clcp-v1 and MG753774.1. [file 12864_2021_7598_MOESM1_ESM.docx]

**Additional file 1:**

| **Table S1. Structural variations between Clcp-v1 and MG753774.1.** | | | | |  |
| --- | --- | --- | --- | --- | --- |
| **Variant type** | **Start in Clcp-v1** | **End in Clcp-v1** | **Gap length in Clcp-v1** | **Gap length in MG753774.1** | **Length difference** |
| Insertion | 10667 | 11690 | 1024 | 0 | 1024 |
| Deletion | 26954 | 26953 | 0 | -75 | 75 |
| Insertion | 40529 | 43860 | 3332 | 0 | 3332 |
| Insertion | 43929 | 44839 | 911 | 0 | 911 |
| Deletion | 80549 | 80526 | -22 | 707 | -729 |
| Insertion | 81812 | 84659 | 2848 | -1 | 2849 |
| Deletion | 104045 | 104041 | -3 | 32 | -35 |
| Deletion | 121399 | 121458 | 60 | -60 | 120 |

| **Table S2. Indels between Clcp-V1 and MG753774.1.** | | | | |
| --- | --- | --- | --- | --- |
| **Position in Clcp-v1** | **Position in MG753774.1** | **Sequence in Clcp-V1** | **Sequence in MG753774.1** | **Related to tandem repeat sequence** |
| 4330 | 6268 | A | A |  |
| 6129 -6139 | 8066 | TTATTATGCTT | . |  |
| 31491 | 32320-32325 | . | GGTCAG | Yes |
| 37011 | 37844 | A | . |  |
| 39464 | 40296 | T | . |  |
| 47960 | 44550-44551 | . | AA |  |
| 48199-48204 | 44789 | TCTGGG | . | Yes |
| 48338-48343 | 44922 | TCTGGT | . | Yes |
| 50139 | 46717 | A | . |  |
| 54003-54010 | 50580 | ATTATGCT | . |  |
| 54017 | 50586 | A | . |  |
| 56486 | 53056 | . | G |  |
| 88143 | 82596 | . | T |  |
| 92205 | 86659-86670 | . | TAACCATAACCA |  |
| 93199-93210 | 87663 | CACGAAGTACTT | . | Yes |
| 97391 | 91845-91854 | . | AAGAAAATTG | Yes |
| 103483 -103486 | 97945 | ATAT | . |  |
| 109601 | 104096 | . | T |  |
| 118495 | 112989 | ATT | . |  |

| **Table S3. Single nucleotide variants (SNVs) between Clcp-V1 and MG753774.1.** | | | |
| --- | --- | --- | --- |
| **Position in**  **Clcp-v1** | **Position in**  **MG753774.1** | **Sequence in**  **Clcp-V1** | **Sequence in**  **MG753774.1** |
| 374 | 2313 | C | T |
| 1947 | 3886 | A | G |
| 7382 | 9309 | C | T |
| 12162 | 13065 | C | A |
| 29211 | 30039 | A | C |
| 33604 | 34438 | C | A |
| 34236 | 35070 | A | T |
| 34884 | 35718 | G | T |
| 34999 | 35833 | G | T |
| 37306 | 38139 | T | C |
| 37570 | 38403 | C | T |
| 37585 | 38418 | A | G |
| 37765 | 38598 | T | C |
| 38028 | 38861 | T | C |
| 38507 | 39340 | C | T |
| 48188 | 44779 | T | C |
| 48195 | 44786 | C | A |
| 48452 | 45031 | T | C |
| 50182 | 46760 | G | A |
| 54093 | 50662 | A | C |
| 55915 | 52484 | C | G |
| 93016 | 87481 | A | T |
| 93725 | 88178 | C | T |
| 97252 | 91705 | T | C |
| 97253 | 91706 | G | A |
| 110322 | 104817 | T | C |
| 115019 | 109514 | C | T |
| 115637 | 110132 | G | T |
| 115859 | 110354 | G | A |
| 122044 | 116416 | A | T |
| 122549 | 116921 | G | C |
| 123147 | 117519 | C | G |
| 123202 | 117574 | A | G |
