# Supplementary material for: Identification of polycistronic transcriptional units and non-canonical introns in green algal chloroplasts based on long-read RNA sequencing data
Source: BMC Genomics. 2021 Apr 23;22:298. doi: 10.1186/s12864-021-07598-y (PMC8063479; doi:10.1186/s12864-021-07598-y)
Supplement: Supplementary file 2 — Additional file 2: Figure S1. Confirmation of exon and intron boundaries of intron-containing genes in chloroplast genome of C. lentillifera. Figure S2. Correction of misleading introns in chloroplast genome of C. lentillifera. [file 12864_2021_7598_MOESM2_ESM.docx]

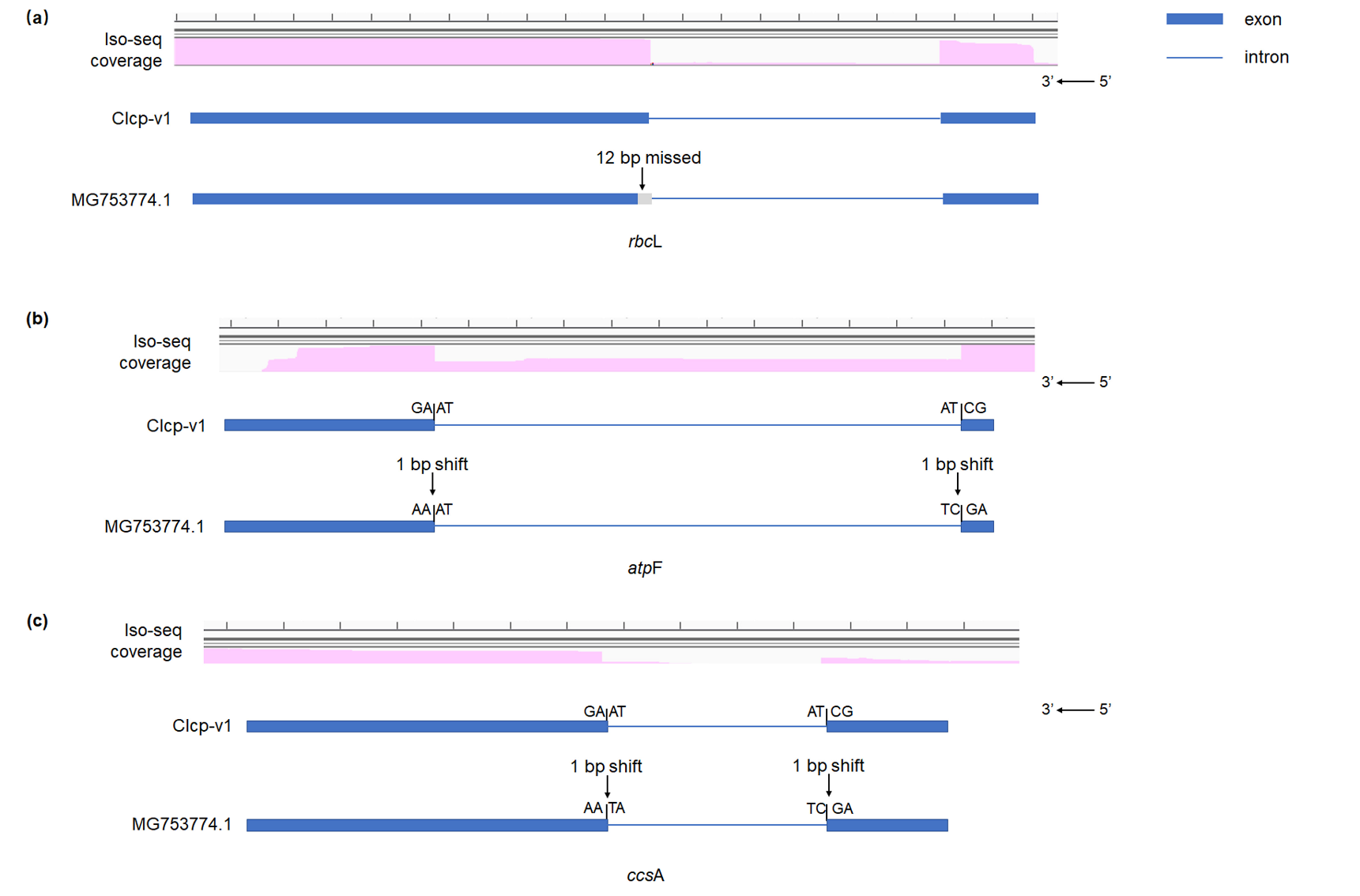


Figure S1. Confirmation of exon and intron boundaries of intron-containing genes in chloroplast genome of *C. lentillifera*.


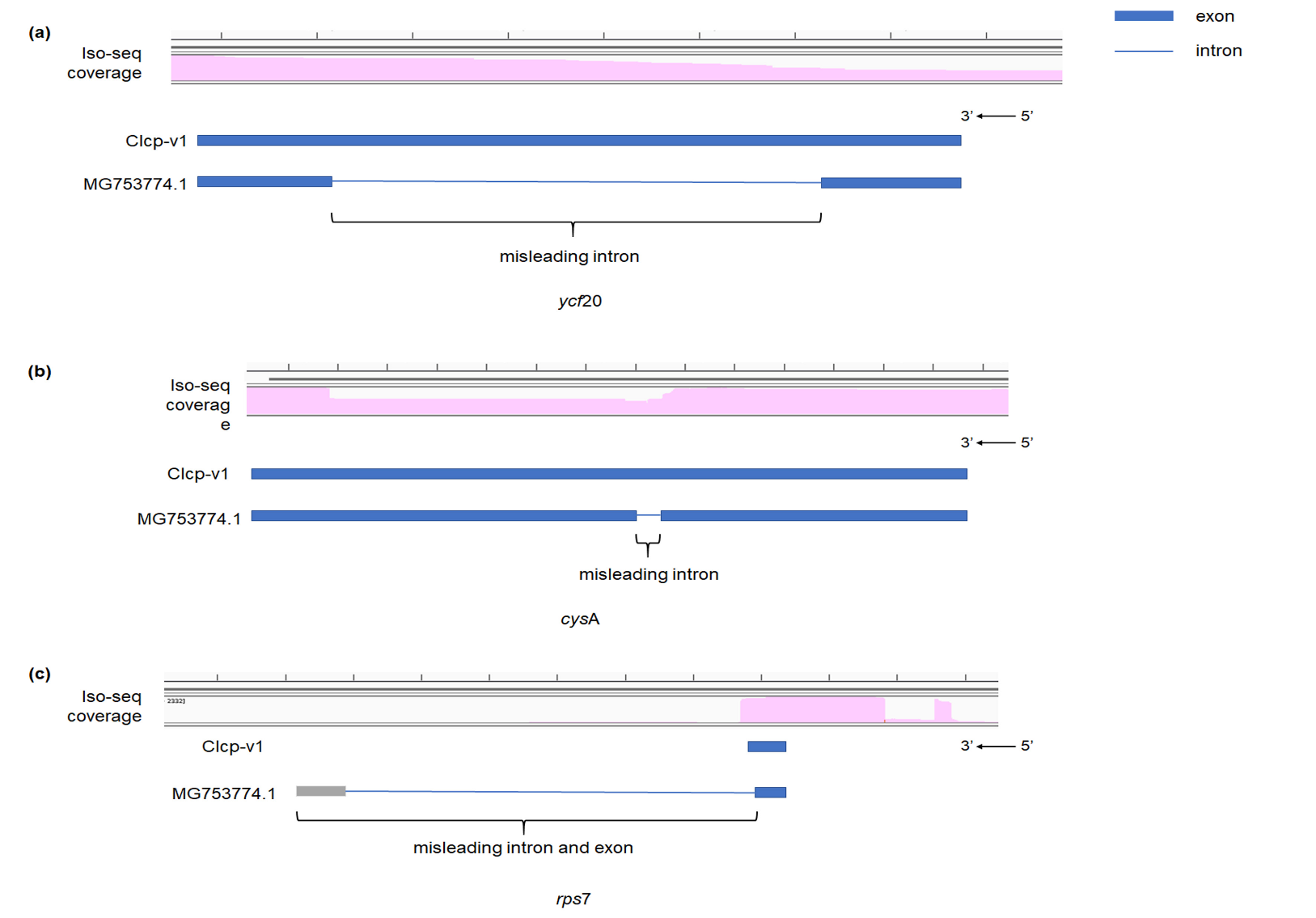


Figure S2. Correction of misleading introns in chloroplast genome of *C. lentillifera*.
