# Supplementary material for: Identification of polycistronic transcriptional units and non-canonical introns in green algal chloroplasts based on long-read RNA sequencing data
Source: BMC Genomics. 2021 Apr 23;22:298. doi: 10.1186/s12864-021-07598-y (PMC8063479; doi:10.1186/s12864-021-07598-y)
Supplement: Supplementary file 5 — Additional file 5: Figure S3. RT-PCR validation of four PTUs in chloroplast genome of C. lentillifera. Full-length blots/gels are presented in Supplementary Figure S4. Figure S4. The uncropped full-length gel for RT-PCR validation of PTUs in Figure S3. [file 12864_2021_7598_MOESM5_ESM.docx]

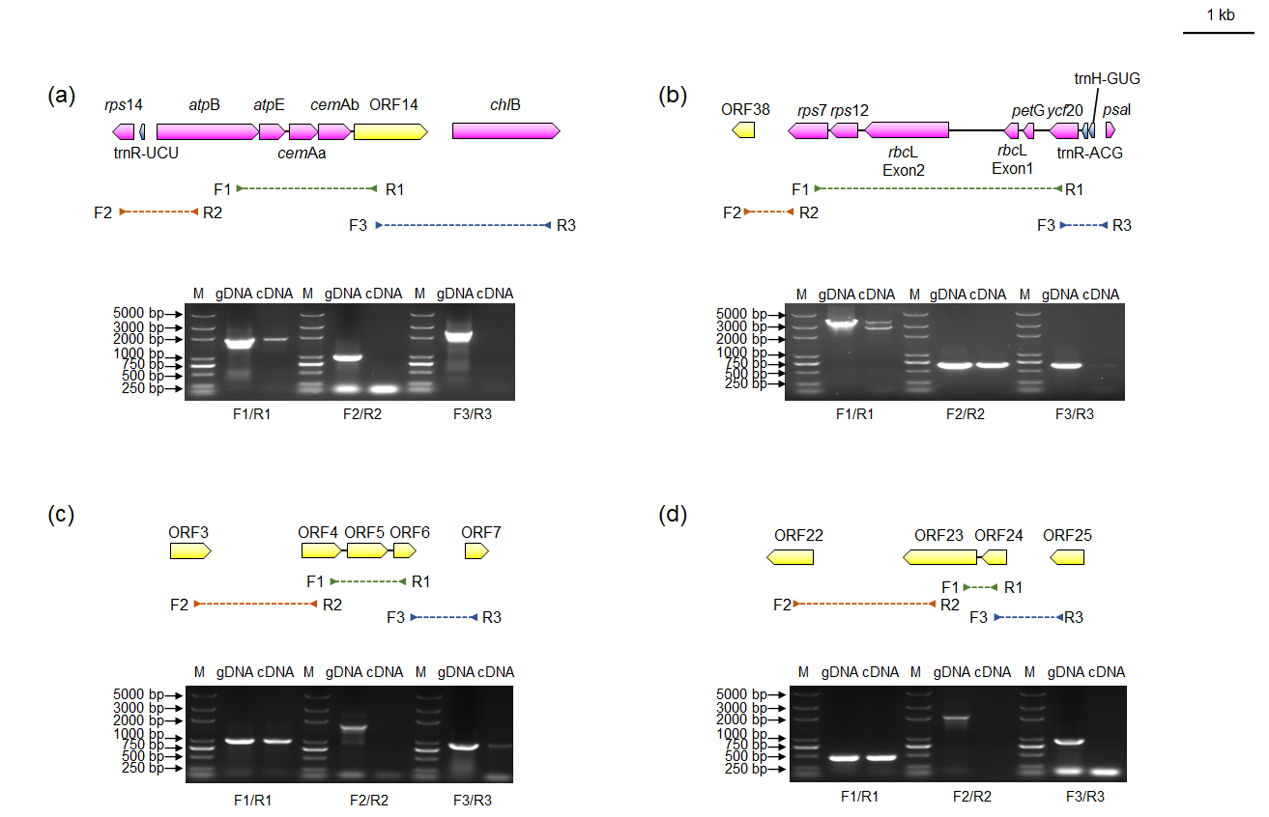


Figure S3. RT-PCR validation of four PTUs in chloroplast genome of *C. lentillifera*. Genes within the PTU are linked by black lines. For each PTU, three pairs of primers span the PTU or the adjacent gene to the gene at the 5’ or 3’end of PTU were shown as arrows with different color. gDNA (genomic DNA) was used as positive control. The two bands of F1/R1 that amplified from cDNA sample were two isoforms with lengths of 3.5 and 2.7 kb in (b), due to an intron retention event of *rbc*L.

Figure S4. The uncropped full-length gel for RT-PCR validation of PTUs in Figure S3. Amplicons from different primer pairs of PTUs are labelled.
