# Supplementary material for: Identification of polycistronic transcriptional units and non-canonical introns in green algal chloroplasts based on long-read RNA sequencing data
Source: BMC Genomics. 2021 Apr 23;22:298. doi: 10.1186/s12864-021-07598-y (PMC8063479; doi:10.1186/s12864-021-07598-y)
Supplement: Supplementary file 7 — Additional file 7: Figure S5. Iso-seq reads alignment of the three fragmented gene in chloroplast genome of C. lentillifera. Figure S6. The uncropped full-length gels for transcriptional characteristics analysis of fragmented genes in Fig. 4. [file 12864_2021_7598_MOESM7_ESM.docx]

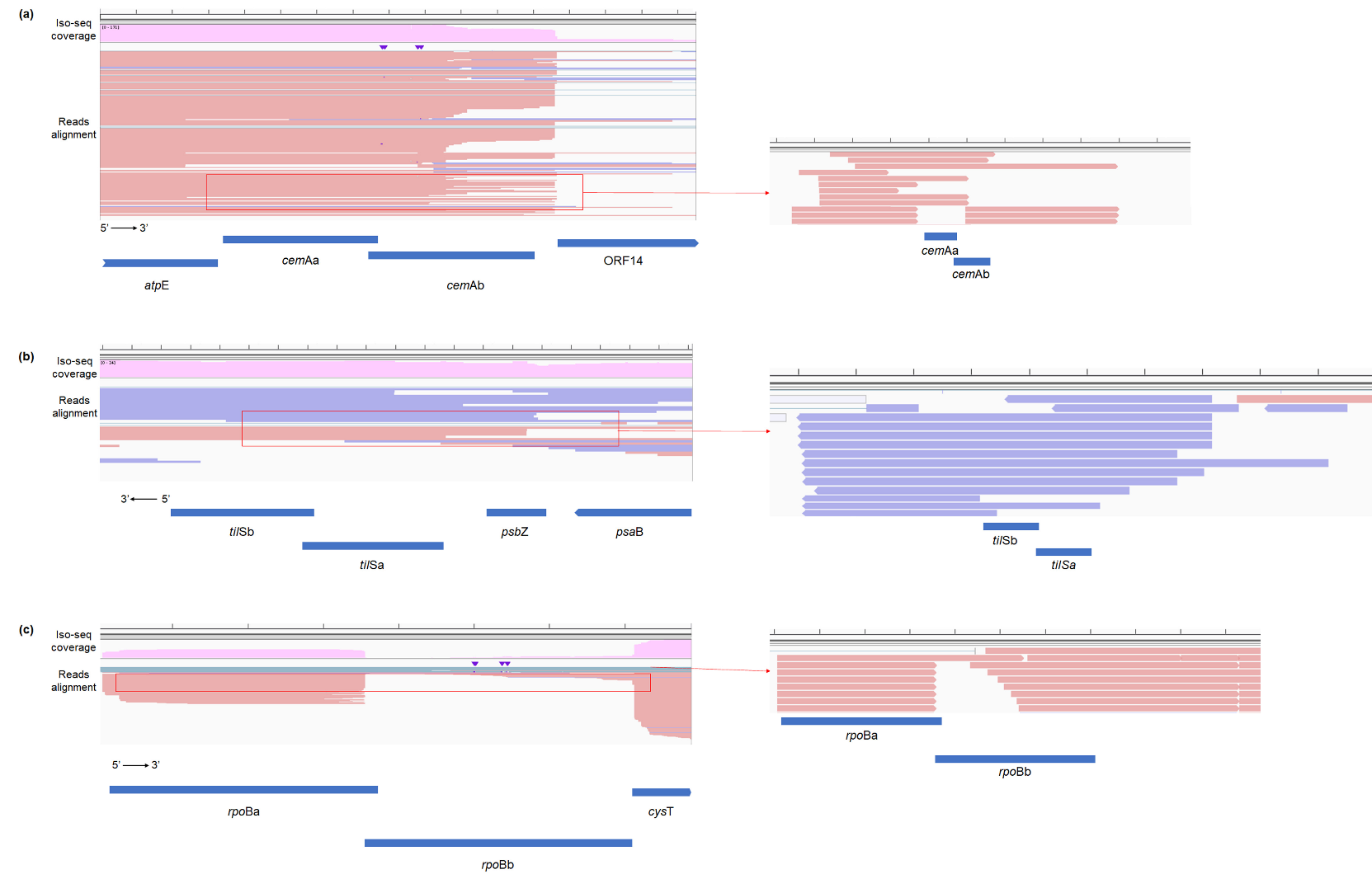


Figure S5. Iso-seq reads alignment of the three fragmented gene in chloroplast genome of *C. lentillifera*.

Figure S6. The uncropped full-length gels for transcriptional characteristics analysis of fragmented genes in Fig. 4. Each lane is labelled according to the name of the sample in the cropped gels. To keep the conciseness of the picture in Fig. 4, a few additional amplicons which are not shown in Fig. 4 are labelled as blue text. Amplicons of other genes (not related to this study) are also indicated.
