# Supplementary material for: Identification of polycistronic transcriptional units and non-canonical introns in green algal chloroplasts based on long-read RNA sequencing data
Source: BMC Genomics. 2021 Apr 23;22:298. doi: 10.1186/s12864-021-07598-y (PMC8063479; doi:10.1186/s12864-021-07598-y)
Supplement: Supplementary file 9 — Additional file 9: Figure S7. Comparison of multi-sequence alignments between original annotation sequences in the database (a) and amino acid sequences that deduced by adjusting the exon-intron boundaries (b) of atpF. Figure S8. Comparison of multi-sequence alignments between original annotation sequences in the database (a) and amino acid sequences that deduced by adjusting the exon-intron boundaries (b) of ccsA (partial). [file 12864_2021_7598_MOESM9_ESM.docx]

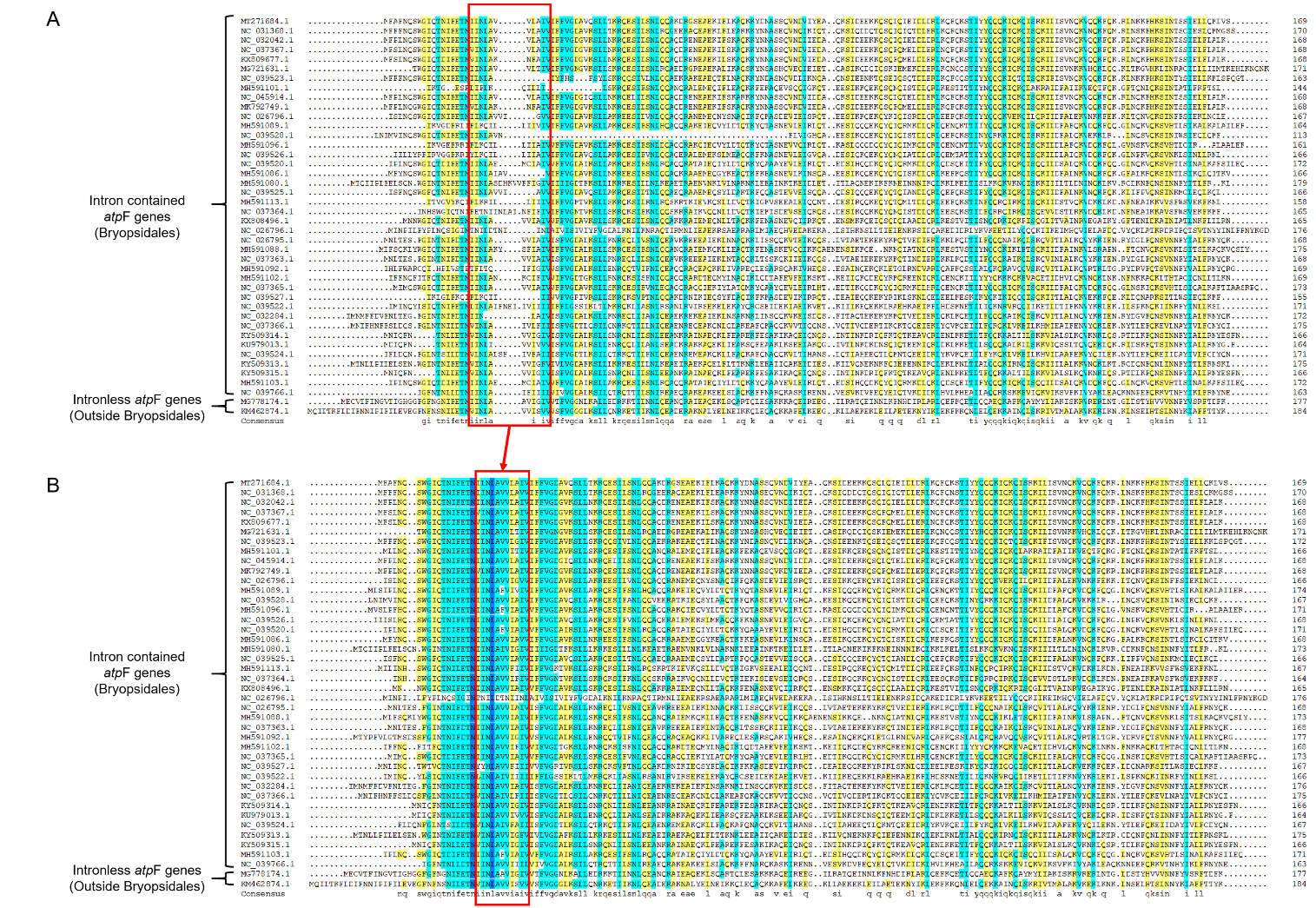


Figure S7. Comparison of multi-sequence alignments between original annotation sequences in the database (a) and amino acid sequences that deduced by adjusting the exon-intron boundaries (b) of *atp*F.


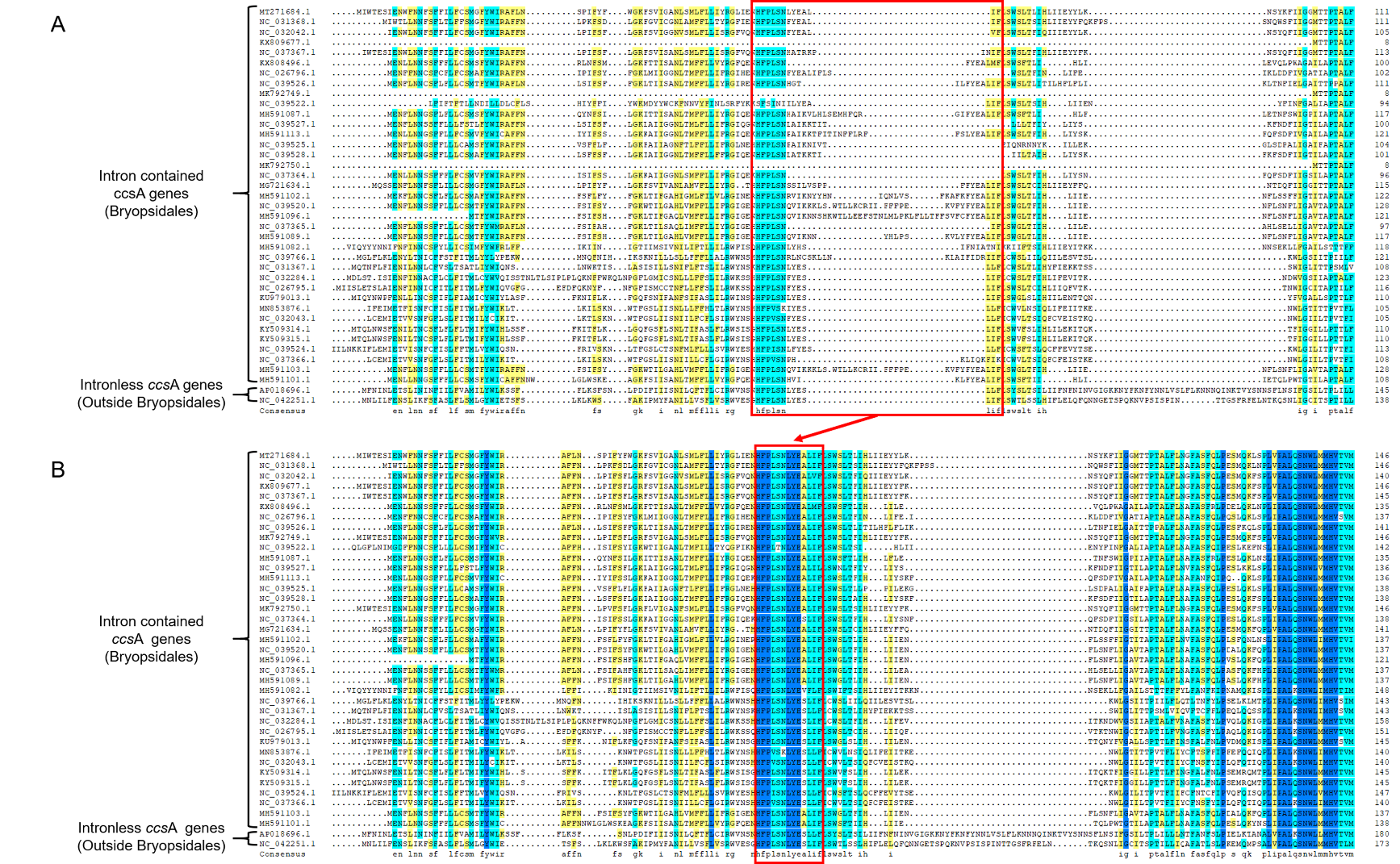


Figure S8. Comparison of multi-sequence alignments between original annotation sequences in the database (a) and amino acid sequences that deduced by adjusting the exon-intron boundaries (b) of *ccs*A (partial).
