# Supplementary material for: Identification of polycistronic transcriptional units and non-canonical introns in green algal chloroplasts based on long-read RNA sequencing data
Source: BMC Genomics. 2021 Apr 23;22:298. doi: 10.1186/s12864-021-07598-y (PMC8063479; doi:10.1186/s12864-021-07598-y)
Supplement: Supplementary file 10 — Additional file 10: Figure S9. Locations of the top five motifs identified from the 40 atpF and 37 ccsA intron sequences. Motif 1 to 5 were represented as block diagrams with different colors as shown in the figure. Figure S10. Sequence logos of domain V from atpF, ccsA, and group II introns (rbcL and psbA), respectively. The two catalytically important elements (the catalytic triad and the bulge region) in domain V were highlighted. [file 12864_2021_7598_MOESM10_ESM.docx]

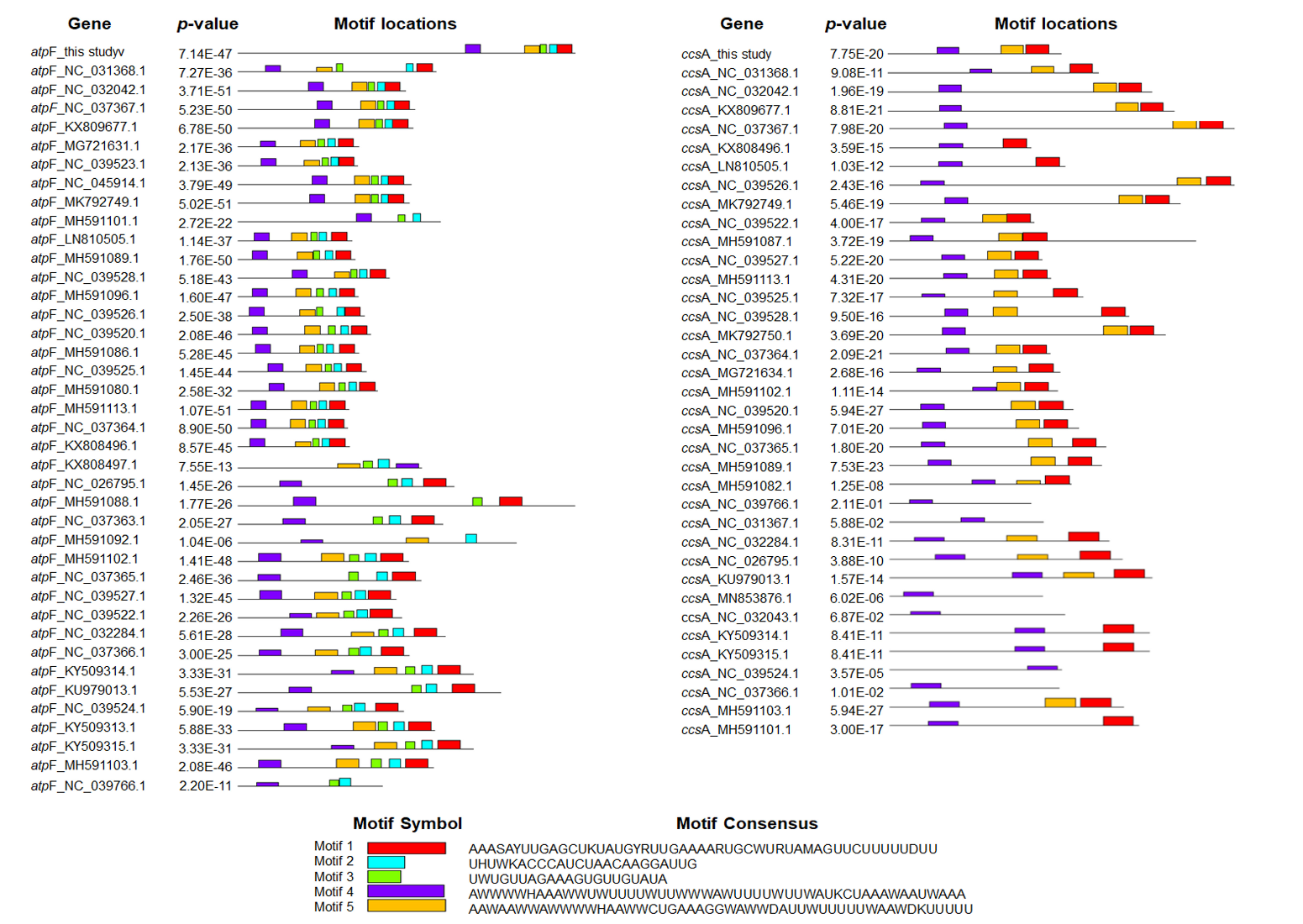


Figure S9. Locations of the top five motifs identified from the 40 *atp*F and 37 *ccs*A intron sequences. Motif 1 to 5 were represented as block diagrams with different colors as shown in the figure.


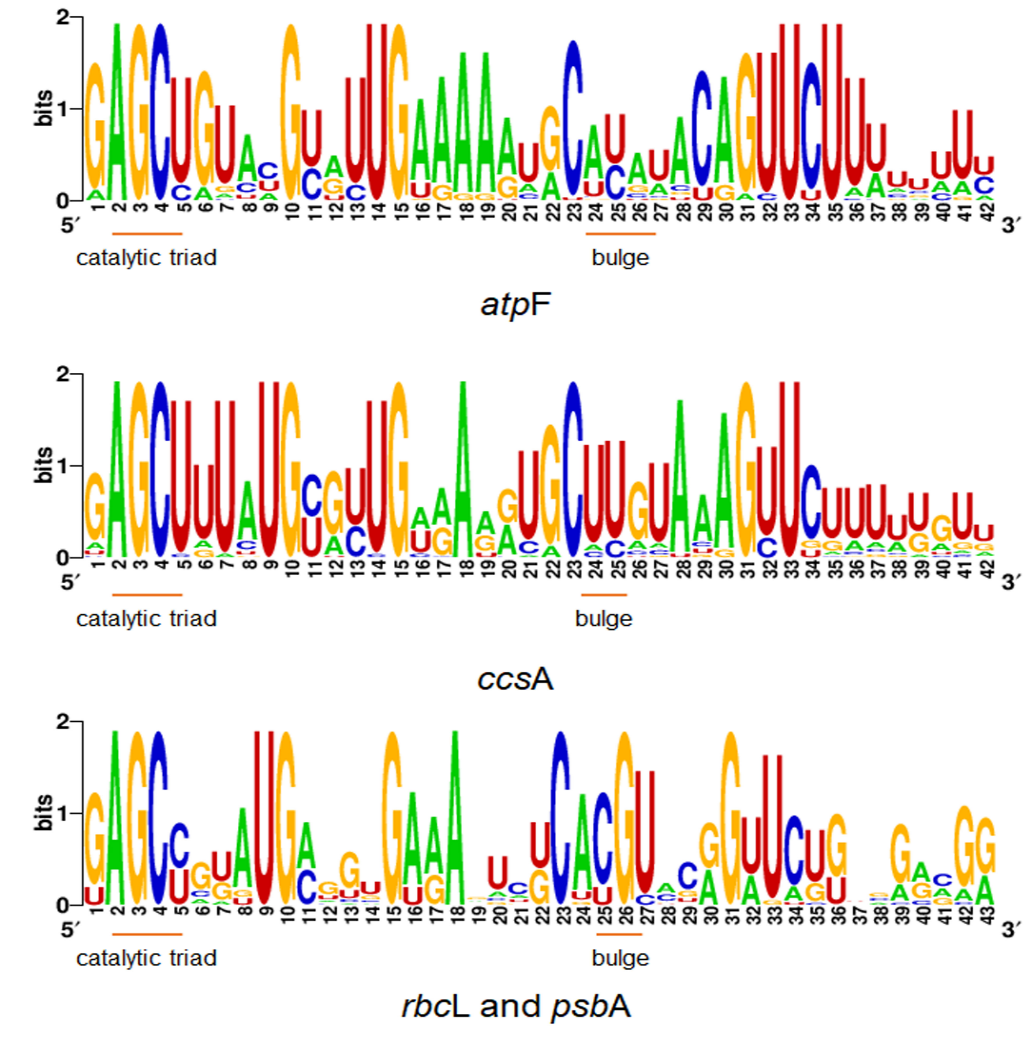


Figure S10. Sequences logos of domain V from *atp*F, *ccs*A, and group II introns (*rbc*L and *psb*A), respectively. The two catalytically important elements (the catalytic triad and the bulge region) in domain V were highlighted.
